# Characterization of two closely related citrus cultivars using UPLC-ESI-MS/MS-based widely targeted metabolomics

**DOI:** 10.1371/journal.pone.0254759

**Published:** 2021-07-20

**Authors:** Fu Wang, Lin Chen, Shiwei Chen, Hongping Chen, Youping Liu

**Affiliations:** 1 Department of Pharmacy, Standardization Education Ministry Key Laboratory of Traditional Chinese Medicine, Chengdu University of TCM, Chengdu, Sichuan, China; 2 Food & Drugs Authority of Nanchong, Nanchong, Sichuan, China; Institute for Biological Research, University of Belgrade, SERBIA

## Abstract

Citrus cultivars are widely spread worldwide, and some of them only differ by specific mutations along the genome. It is difficult to distinguish them by traditional morphological identification. To accurately identify such similar cultivars, the subtle differences between them must be detected. In this study, UPLC-ESI-MS/MS-based widely targeted metabolomics analysis was conducted to study the chemical differences between two closely related citrus cultivars, *Citrus reticulata* ‘DHP’ and *C*. *reticulata* ‘BZH’. Totally 352 metabolites including 11 terpenoids, 35 alkaloids, 80 phenolic acids, 25 coumarins, 7 lignans, 184 flavonoids and 10 other compounds were detected and identified; Among them, 15 metabolites are unique to DHP and 16 metabolites are unique to BZH. Hierarchical cluster analysis (HCA), principal component analysis (PCA), and orthogonal signal correction and partial least squares-discriminant analysis (OPLS-DA) can be used to clearly discriminate between DHP and BZH. 93 metabolites including 36 down-regulated and 57 up-regulated are significantly different in DHP and BZH. They are mainly involved in the biosynthesis of flavonoids, flavones, flavonols, and isoflavonoids. In addition, the relative content levels of flavonoids, alkaloids, and terpenoids are much higher in the peel of DHP than that of BZH, the presence of which may correlate with the quality difference of the peels. The results reported herein indicate that metabolite analysis based on UPLC-ESI-MS/MS is an effective means of identifying cultivars with different genotypes, especially those that cannot be distinguished based on traditional identification methods.

## Introduction

The *Citrus* genus adapts to new climates, creating diverse genotypes [1]. Among these bud-mutation-derived varieties, the *Citrus reticulata* ‘DHP’ and *C*. *reticulata* ‘BZH’ both grow in Sichuan Province of China [2]. For more than 2000 years, the peel of DHP has been widely used to treat dyspepsia [3], and it is commonly believed that the longer this peel is stored, the higher its quality and efficiency [4]. However, the BZH peel has no therapeutic effects and is generally used as a tea or seasoning. The similar morphology and genomic DNA sequences of DHP and BZH peels impede the distinction between the two cultivars using traditional identification methods [5]. As a result, BZH is often mistaken for DHP and sold as a therapeutic material [6].

Plant secondary metabolites are natural products that have small sizes and high structural diversity [7]. The metabolites in a particular organism may be quantified using metabolomics. Metabolomics is the study of chemical processes involving metabolites, the small molecule substrates, intermediates and products of cell metabolism. Systems biology and functional genomics try to integrate genomics, transcriptomic, proteomic, and metabolomic information to provide a better understanding of cellular biology [8]. Traditional liquid chromatography-mass spectrometry methods include targeted metabolomics and non-targeted metabolomics. Targeted metabolomics can only detect a few known metabolites qualitatively and quantitatively, however, it has high sensitivity and accuracy in quantification [9]. By contrast, the non-targeted metabolomics method can simultaneously detect hundreds or even thousands of metabolites (including known and unknown metabolites). The drawback is that its sensitivity is lower, and the qualitative and quantitative accuracy is relatively poor [10]. In view of the advantages and disadvantages of the above two methods, a widely-targeted metabolomics analysis method has been developed in recent years. It can simultaneously qualitatively and quantitatively analyze hundreds of known and unknown metabolites [11]. This analytical method has the same sensitivity and quantitative accuracy as the targeted method. Meanwhile, it offers the advantages of wider coverage and has been successfully applied to studies of metabolites in plant wheat [12], corn [13], tomato [14], grape [15] and other crops [16–19]. However, this method is complicated in sample processing and detection process, and relies on self-built databases. The self-built database used in this paper contains more than 5000 compounds, which can satisfy the study of metabolites in the samples analyzed. Meanwhile, the establishment of the detection method and the stability of the instruments are very important for the accuracy and reproducibility of the test results. When using high throughput detection technology to study metabolomics, the changes of instrument performance will cause deviations in analysis results over time. In order to monitor the performance of the instruments during the tests, some researchers put forward the order of random arrangement of samples and prepared quality control samples (QC sample) [20]. QC sample can be a mixed reference substance of representative substances or a pooled QC sample of uniform mixing of samples to be analyzed [21]. When using QC samples for quality control, it is necessary to enter 5–10 needles before formally starting the test to stabilize the state of the instrument, and to insert QC samples randomly in the middle of the analysis sequence to monitor the stability of the whole analysis sequence. Recently, widely-targeted metabolomics analyses have been successfully applied in the identification of plant species with similar morphologies. For example, Duan et al [22]. have used this technique to differentiate between medicinal *Glycyrrhiza* species and their hybrids. Consequently, metabolomics may be an effective method to differentiate between citrus cultivars.

In this study, a widely targeted metabolomic method was employed to analyze the types and relative contents of metabolites of two closely related citrus cultivars DHP and BZH. Hierarchical cluster analysis (HCA), principal component analysis (PCA), and orthogonal signal correction and partial least squares-discriminant analysis (OPLS-DA) were used to discriminate between DHP and BZH. The relative contents of different kinds of metabolites were compared with metabolite ion intensity. The obtained results provide a reference for the identification of closely related cultivars.

## Materials and methods

### Plant materials

The mature tangerine pericarp of *C*. *reticulata* ‘DHP’ and *C*. *reticulata* ‘BZH’ were collected from Wugui village, Qingquan town, Qingjiang district, Chengdu city, Sichuan province on December 11th, 2020 (Lat. 30°88′ N, 104°32′ E, Alt. 423 m). For each sample, the mixed peels were collected from the same six adult trees in the field and had three independent biological replicates. The peel samples were frozen in liquid nitrogen immediately after collection and stored at -80°C before use.

### Sample preparation and extraction

The freeze-dried citrus peel samples were crushed for 1.5 min using a mixer mill (MM400, Retsch, Germany) equipped with a zirconia bead and operated at 30 Hz. Subsequently, 100 mg of the crushed powder was weighed and extracted overnight at 4°C with 1.0 mL 70% aqueous methanol. Afterwards, each sample was centrifuged at 10,000 g for 10 min, then the extracts were absorbed (CNWBOND Carbon-GCB SPE Cartridge, 250 mg, 3 mL; ANPEL, Shanghai, China) and filtered (SCAA-104, 0.22 μm pore size; ANPEL, Shanghai, China) before LC-MS analysis.

### UPLC conditions

The sample extracts were analyzed using LC-ESI-MS/MS (UPLC, Shim-pack UFLC SHIMADZU CBM30A system; MS, Applied Biosystems 6500 QTRAP, America), based on the method published by Chen et al [23]. The sample components were separated on a Waters ACQUITY UPLC HSS T3 C18 column (1.8 μm, 2.1 mm × 100 mm; Waters, America) using a mixture of water (0.04% acetic acid) and acetonitrile (0.04% acetic acid) as mobile phase. The gradient program was set to 100:0 v/v water:acetonitrile at 0 min, 5:95 v/v at 11.0 min, 5:95 v/v at 12.0 min, 95:5 v/v at 12.1 min, and 95:5v/v at 15.0 min. The chromatographic analyses were performed at 40°C and 0.40 mL/min flow rate, and the injection volume was set to 2 μL. The effluent was alternately connected to an ESI-triple quadrupole-linear ion trap (Q TRAP)-MS [24].

### ESI-Q TRAP-MS/MS

LIT and triple quadrupole (QQQ) scans were recorded on a triple quadrupole-linear ion trap mass spectrometer (Q TRAP). The API 6500 Q TRAP LC/MS/MS system was equipped with an ESI TurboIonSpray interface, and it was operated on positive ion mode. The Analyst 1.6.3 software (AB Sciex) was used to control the analytical system and analyze the acquired data. Operation parameters of the ESI source are as follows: turbo spray ion source, 500°C source temperature, 5500 V ion spray voltage (IS), 55 psi ion source gas I (GSI), 60 psi ion source gas II (GSII), 25 psi curtain gas (CUR), and high collision gas (CAD). Instrument tuning and mass calibration were performed using 10 and 100 μmol/L polypropylene glycol solutions in QQQ and LIT modes, respectively. The QQQ scans were acquired via MRM experiments using 5 psi nitrogen collision gas. The DP and CE of individual MRM transitions were optimized, and a specific set of the transitions was monitored during each period based on the eluted metabolites [25, 26].

### Principle of qualitative and quantitative analysis of metabolites

Based on the self-built MWDB database [27] (Metware Biotechnology Co., Ltd. Wuhan, China) and the public database of metabolite information, the metabolites in different samples were qualitatively and quantitatively analyzed using mass spectrometry. The analysis removed the isotope signal, a repetitive signal containing K+ ions, Na+ ions, NH4+ ions, and a repetitive signal of fragment ions with larger molecular weight themselves. The characteristic ions of each metabolite were detected by the triple quadrupole rod, and the signal strengths of these ions were measured by the detector. The spectroscopic and chromatographic data were manipulated using the MultiQuant software. The quantification of metabolites was completed by multi-response monitoring mode (MRM) analysis of triple quadrupole mass spectrometry. On the MRM mode, the quadrupole screened the precursor ions of the target substance at frist, then removed the ions corresponding to the other molecular weight substances to eliminate the interference initially. The precursor ions were ionized by the collision chamber and broken down to form fragment ions. The fragment ions were filtered by triple quadrupole to select a characteristic fragment ion needed to eliminate the interference of non-target ions, so that the quantification was more accurate and the repeatability was better.

### Qualitative and quantitative analysis of metabolites

**Fig 1:**
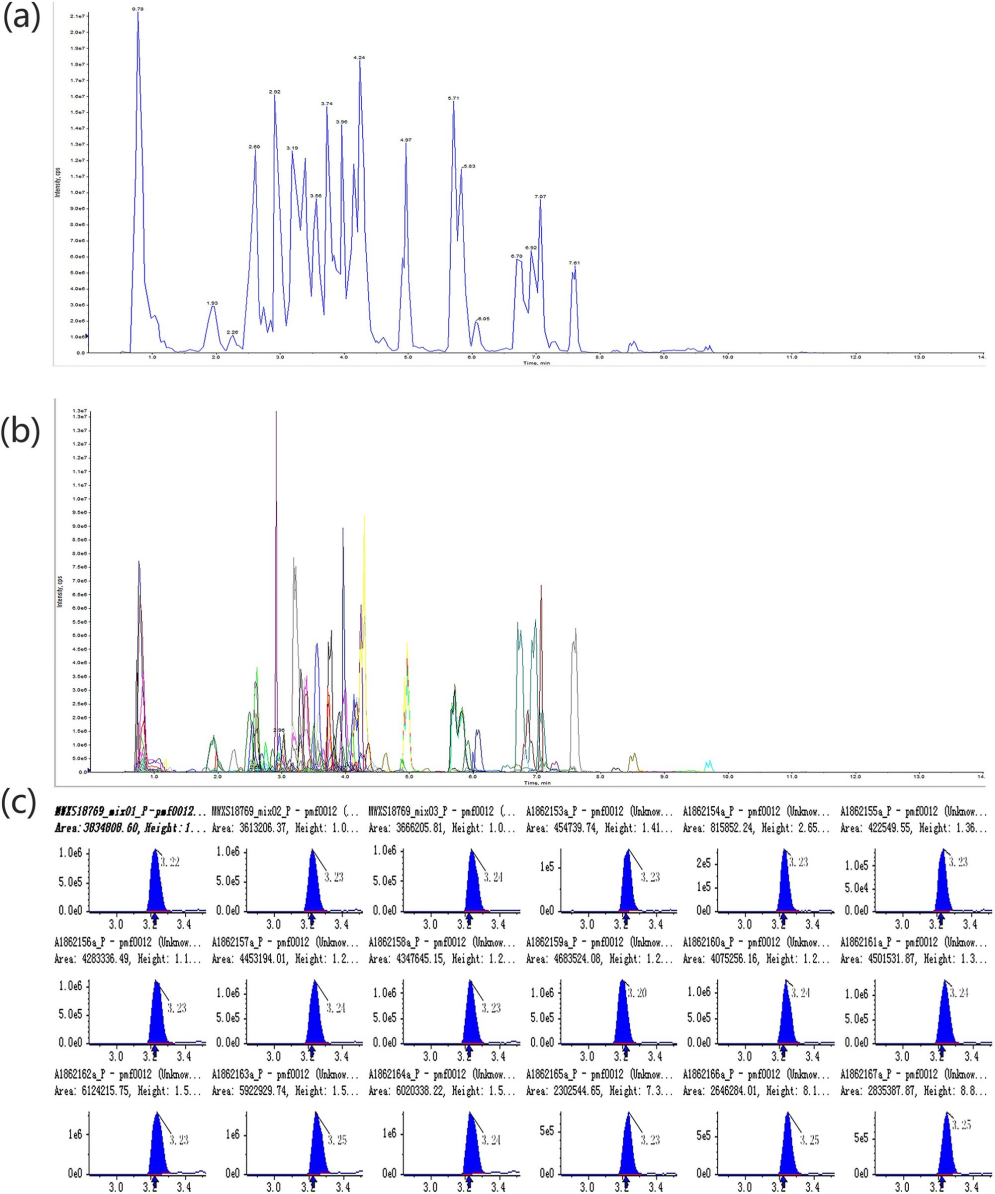
(a) Total Ions Current (TIC) and MRM metabolite detection multi-peak graphs of the mixed-sample (b) Overlapping of total ions flow diagram. (c) Calibration diagrams used for the quantitative analysis of metabolites. The integration correction results are shown for random samples, and the x-axis represents retention time.

The software Analyst 1.6.3 was used to process the mass spectrum data. Total Ions Current (TIC) and MRM metabolite detection multi-peak graphs of the mixed-sample are shown in Fig 1A. The metabolites of the samples were analyzed by mass spectrometry based on the local database. Fig 1B shows the metabolites that can be detected in the samples. Each mass spectrum peak of different colors represents the detection of a metabolite. The characteristic ions of each metabolite were screened by triple quadrupole rod, and the signal intensity of the characteristic ions was obtained by the detector. MultiaQuant software was used to open the mass spectrum file of the samples and to correct and integrate the chromatographic peaks. The area of each chromatographic peak represents the relative content of the metabolites. The peak areas were integrated and corrected, and used to calculate the relative amounts of substances in each sample. To compare the contents of each metabolite in different samples, the mass spectral peaks of the metabolite were calibrated based on the component’s retention time and peak pattern (Fig 1C). The adopted analytical method ensures the accuracy of the qualitative and quantitative data [28].

Principal component analysis (PCA) is a multivariate method that is widely used to summarize data variations, reveal differences between groups, and quantify the variability of samples within the same group [29]. Similarly, OPLS-DA analysis maximizes the variations between groups and is commonly used to screen differential metabolites. The differential metabolites of DHP and HZY were screened based on the fold change and variable importance in project (VIP) values of the OPLS-DA model. Specifically, the metabolites having fold change values ≥2 or ≤0.5 and VIP values ≥1 were identified as differential [30].

### Quality Control (QC) analysis

To prepare the QC sample, we mixed the sample extractions to analyze the repeatability of the samples under the same treatment method. The repeatability of metabolite extractions can be determined by overlapping display analysis of the total ion flow diagram of mass spectrometry of different QC samples. The high stability of the instruments provides an important guarantee for the repeatability and reliability of the data. Overlapping of total ions flow diagram is shown in Fig 1B.

### Bioinformatic analysis

MS raw data (.wiff) files were converted to mzXML format by ProteoWizard, and the data of peak deconvolution, alignment, and integration was processed using R package XCMS (version 3.2). The minfrac and cutoff parameters were set to 0.5 and 0.3, respectively. The in-house MS2 database was used to identify the metabolites. The experiments were performed in triplicate. Cluster analysis, PCA, and OPLS-DA were conducted using R (http://www.r-project.org/), according to previously published methods [31].

## Results

### Profiling

**Fig 2:**
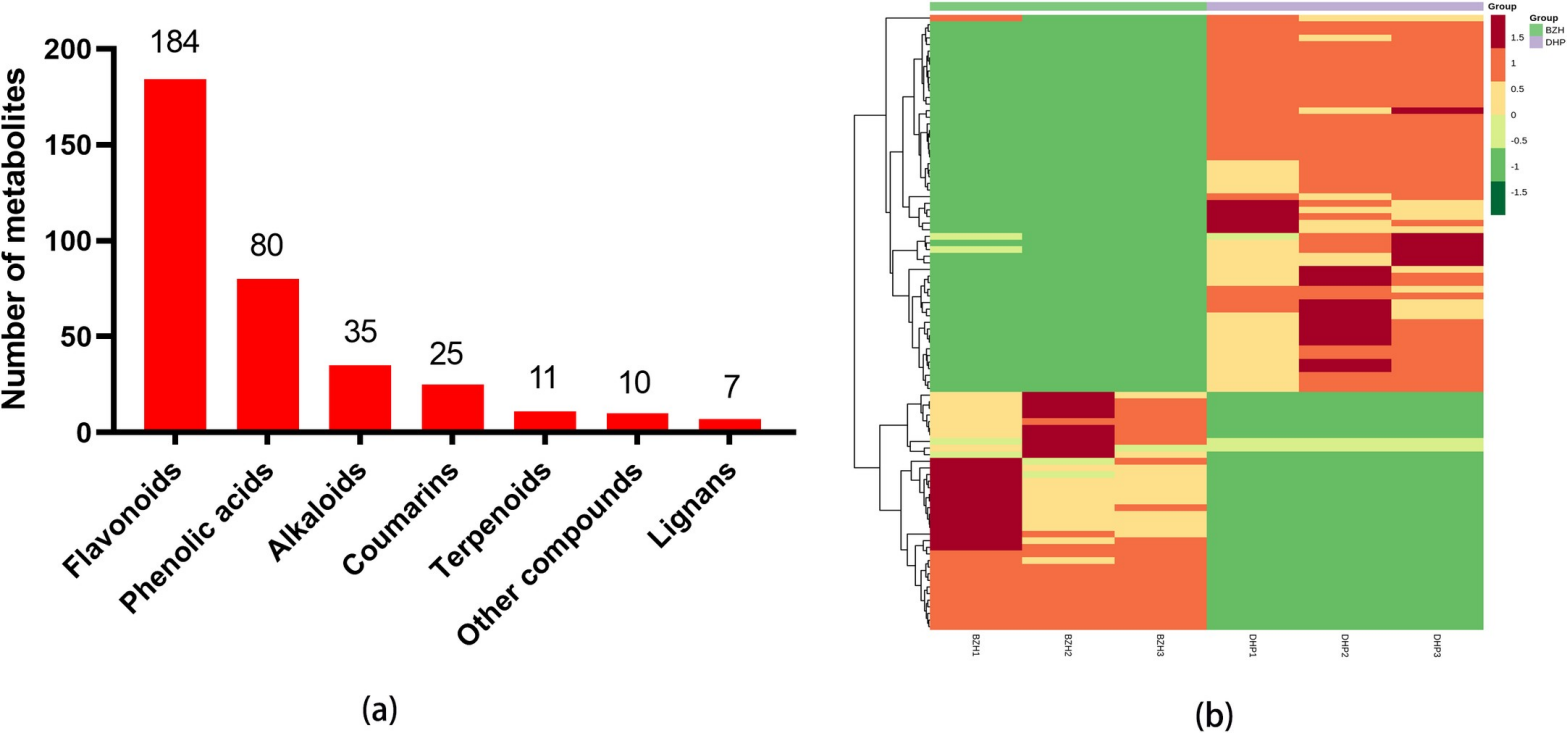
(a) Classification of detected metabolites. (b) Heat-map of differential metabolites in *C*. *reticulata* ‘DHP’ and *C*. *reticulata* ‘BZH’. The up-regulated and down-regulated metabolites were expressed with different shade colors of red and blue, respectively. With the increase in the abundance value, the color of the bar presented from blue to red. When the abundance value was 0, the color of bar was white, as shown in the bar at the upper right.

**Fig 3:**
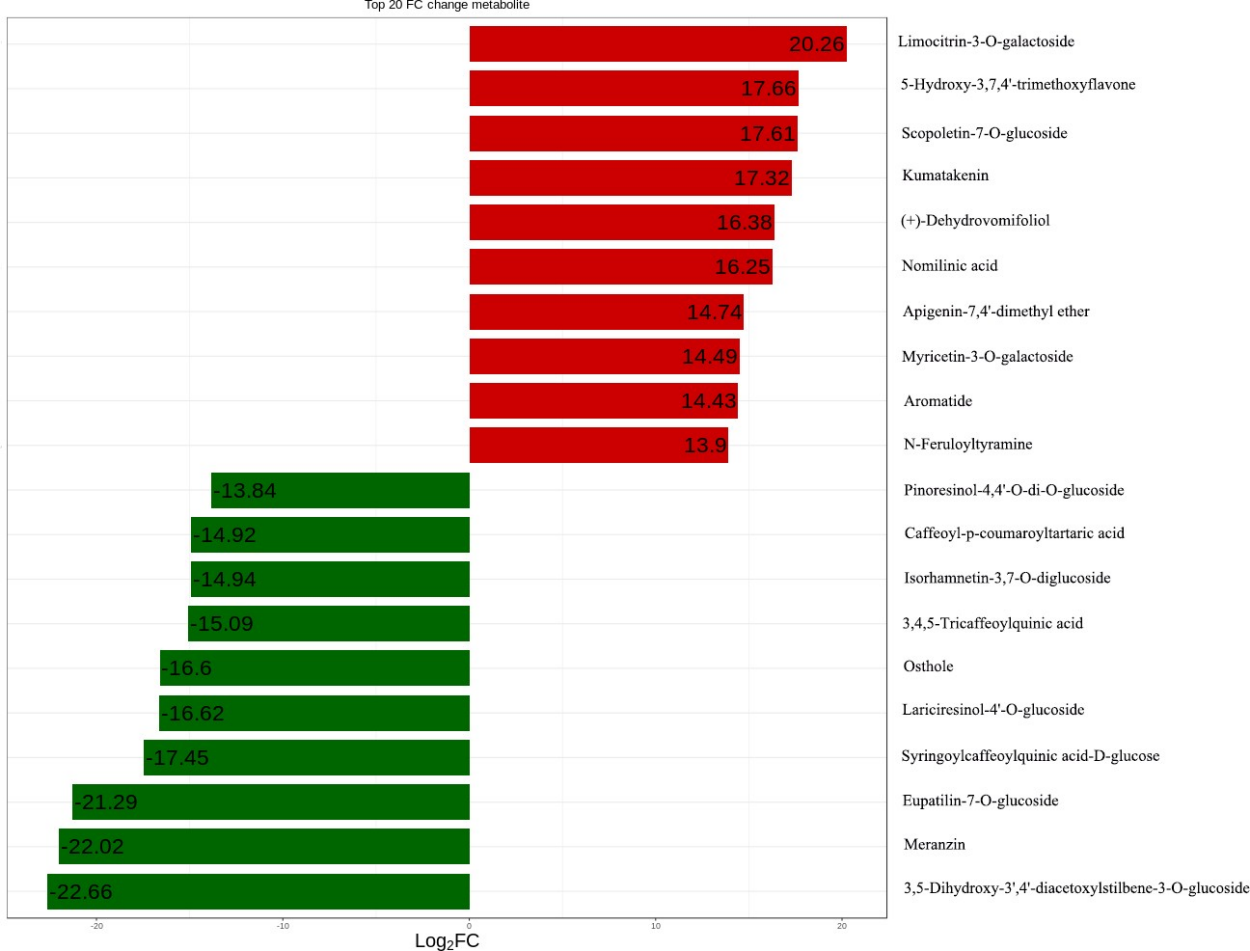
Histogram of fold change of the top 20 metabolites after log2 treatment between *C*. *reticulata* ‘DHP’ and *C*. *reticulata* ‘BZH’.

The metabolites of DHP and BZH peels were identified using UPLC-ESI-MS/MS. Totally 352 metabolites were identified (S1 Table), including 11 terpenoids, 35 alkaloids, 80 phenolic acids, 25 coumarins, 7 lignans, 184 flavonoids and 10 other compounds (Fig 2A). The heat-map presented in Fig 2B shows that among the detected metabolites, 93 are differential in the two cultivars, with 15 unique to DHP and 16 unique to BZH (Tables 1 and 2). These compounds can be used as chemical markers to distinguish between the two citrus cultivars. Clustering analysis of the two samples confirms that they are accurately identified. The fold change of metabolites of the two citrus cultivars were compared and analyzed, the top 20 metabolites with greater changes are shown in Fig 3 after log2 treatment. These compounds are limocitrin-3-O-galactoside, 5-Hydroxy-3,7,4’-trimethoxyflav-one, murrayin, kumatakenin, (+)-Dehydrovomifoliol, nomilinic acid, apigenin-7,4’-dimethyl ether, myricetin-3-O-galactoside, aromatide, n-Feruloyltyramine, pinoresinol-4,4’-O-di-O-glucoside, caffeoyl-p-coumaroyltartaric acid, isorhamnetin-3,7-O-diglucoside, 3,4,5-Tricaffeoylquinic acid, osthole, lariciresinol-4’-O-glucoside, syringoylcaffeoylquinic acid-D-glucose, eupatilin-7-O-glucoside, meranzin, 3,5-Dihydroxy-3’,4’-diacetoxylstilbene-3-O-glucoside.

**Table 1:** Details of potential chemical markers of *C*. *reticulata* ‘BZH’.

ID	Q1 (Da)	Q3 (Da)	Rt (min)	Molecular	Ionization model	Compounds
1	417.2	297.1	4	416.2	[M+H]+	Chrysin C-hexoside
2	637.1	303.4	3.27	637.1	Protonated	Delphinidin O-malonyl-malonylhexoside
3	491.1	287.1	5.09	492.1	[M-H]-	Acetyl-eriodictyol O-hexoside
4	445	283	3.54	446.12	[M-H]-	Glycitin
5	431	269	4.01	432.11	[M-H]-	Genistein 7-O-Glucoside (genistin)
6	433.1	271	2.83	433.1	Protonated	Pelargonidin 3-O-beta-D-glucoside (callistephin chloride)
7	429.1	267	4.59	430.13	[M-H]-	Formononetin 7-O-glucoside (ononin)
8	465.1	300.7	4	464.1	[M-H]-	Gossypitrin
9	301.1	258	6.28	300.06	[M+H]+	Hydroxygenkwanin
10	433.1	271.1	4.21	432.1	[M+H]+	Apigenin 7-O-glucoside (cosmosiin)
11	659.2	331.1	4.5	658.2	[M+H]+	Tricin 4’-O-(syringyl alcohol) ether 5-O-hexoside
12	435.1	303	3.98	434.08	[M+H]+	Quercetin 3-alpha-L-arabinofuranoside (avicularin)
13	285	257	5.08	286.05	[M-H]-	Orobol (5,7,3’,4’-tetrahydroxyisoflavone)
14	595	270.9	2.38	595	Protonated	Pelargonin
15	625.1	445.2	2.47	626.1	[M-H]-	Hesperetin C-hexoside O-hexoside
16	725.2	331.1	4.5	724.2	[M+H]+	Tricin O-rhamnosyl-O-malonylhexoside

Note: Q1 means molecular ion; Q3 means characteristic ion; Rt means retention time; Da means Dalton.

**Table 2:** Details of potential chemical markers of *C*. *reticulata* ‘DHP’.

ID	Q1 (Da)	Q3 (Da)	Rt (min)	Molecular	Ionization model	Compounds
1	519	271	4.23	518	[M+H]+	Apigenin O-malonylhexoside
2	435.1	273	4.22	434.12	[M+H]+	Naringenin 7-O-glucoside (prunin)
3	367.1	149	7.38	368.13	[M-H]-	Curcumin
4	273	229	3.4	274.08	[M-H]-	Afzelechin (3,5,7,4’-Tetrahydroxyflavan)
5	433	287	4.49	432.11	[M+H]+	Kaempferol 3-O-rhamnoside (kaempferin)
6	331	151	5.15	332.05	[M-H]-	Laricitrin
7	479.2	317	4.55	478.15	[M+H]+	Persicoside
8	437.1	275	4.37	436.14	[M+H]+	Phloridzin
9	757.1	433.4	3.1	756.1	[M+H]+	6-C-hexosyl-apigenin O-hexosyl-O-hexoside
10	639.1	331.4	4.07	638.1	[M+H]+	Tricin 5-O-rutinoside
11	625.4	301	2	625.4	Protonated	Peonidin 3, 5-diglucoside chloride
12	771.2	177.1	4.25	770.21	[M+H]+	C-pentosyl-chrysoeriol 7-O-feruloylhexoside
13	565.2	271.1	4.06	564.15	[M+H]+	Apiin
14	697.1	535.1	3.03	697.1	Protonated	Malvidin 3-acetyl-5-diglucoside
15	497.1	331.3	5.75	496.1	[M+H]+	Tricin 4’-O-syringyl alcohol

Note: Q1 means molecular ion; Q3 means characteristic ion; Rt means retention time; Da means Dalton.

### PCA and OPLS-DA analyses of differential flavonoid metabolites

**Fig 4:**
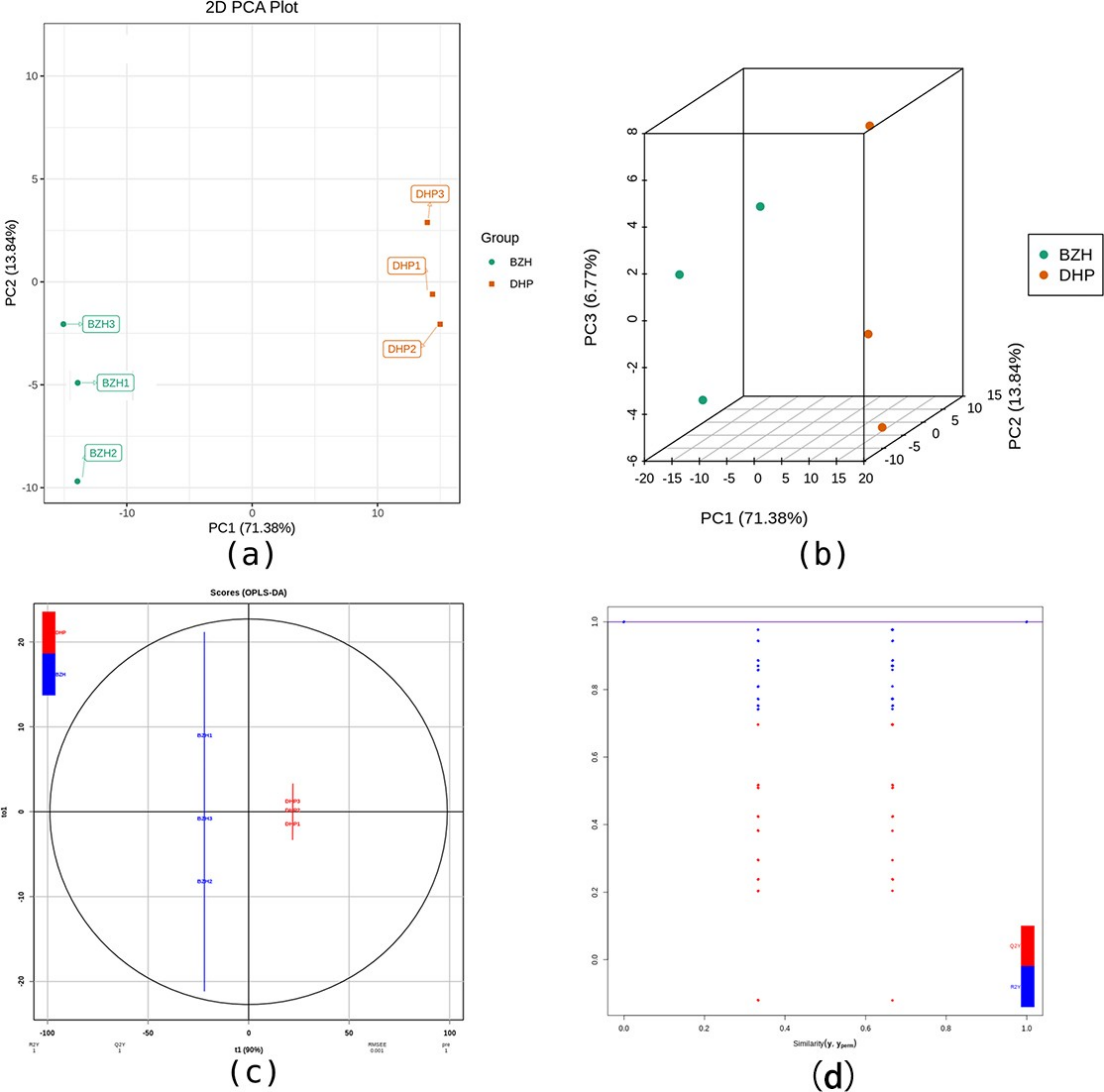
(a,b) PCA and (c,d) OPLS-DA analyses of *C*. *reticulata* ‘DHP’ and *C*. *reticulata* ‘BZH’.

To accurately identify the two citrus cultivars, it is necessary to amplify and detect the subtle differences between them. For this purpose, PCA and OPLS-DA analyses were conducted using the principal differential components. As shown in Fig 4A, the cumulative contribution rate of PC1 and PC2 is 85.22%, with 71.38% attributed to PC1 and 13.84% attributed to PC2 (Fig 4A and 4B). The classification results of PCA show noticeable differences between the DHP and BZH samples. Of the differential metabolites, they were used to establish an OPLS-DA model. The results presented in Fig 4C demonstrate that the R^2^Y and Q^2^ values determined using this model are both 1. Considering that Q^2^ exceeds 0.9 and the red and green dots did not exceed the corresponding line (Fig 4D), and that the OPLS-DA model is stable and reliable and could be used to further screen differential metabolites.

### Differential metabolite screening, functional annotation, and enrichment analysis

**Fig 5:**
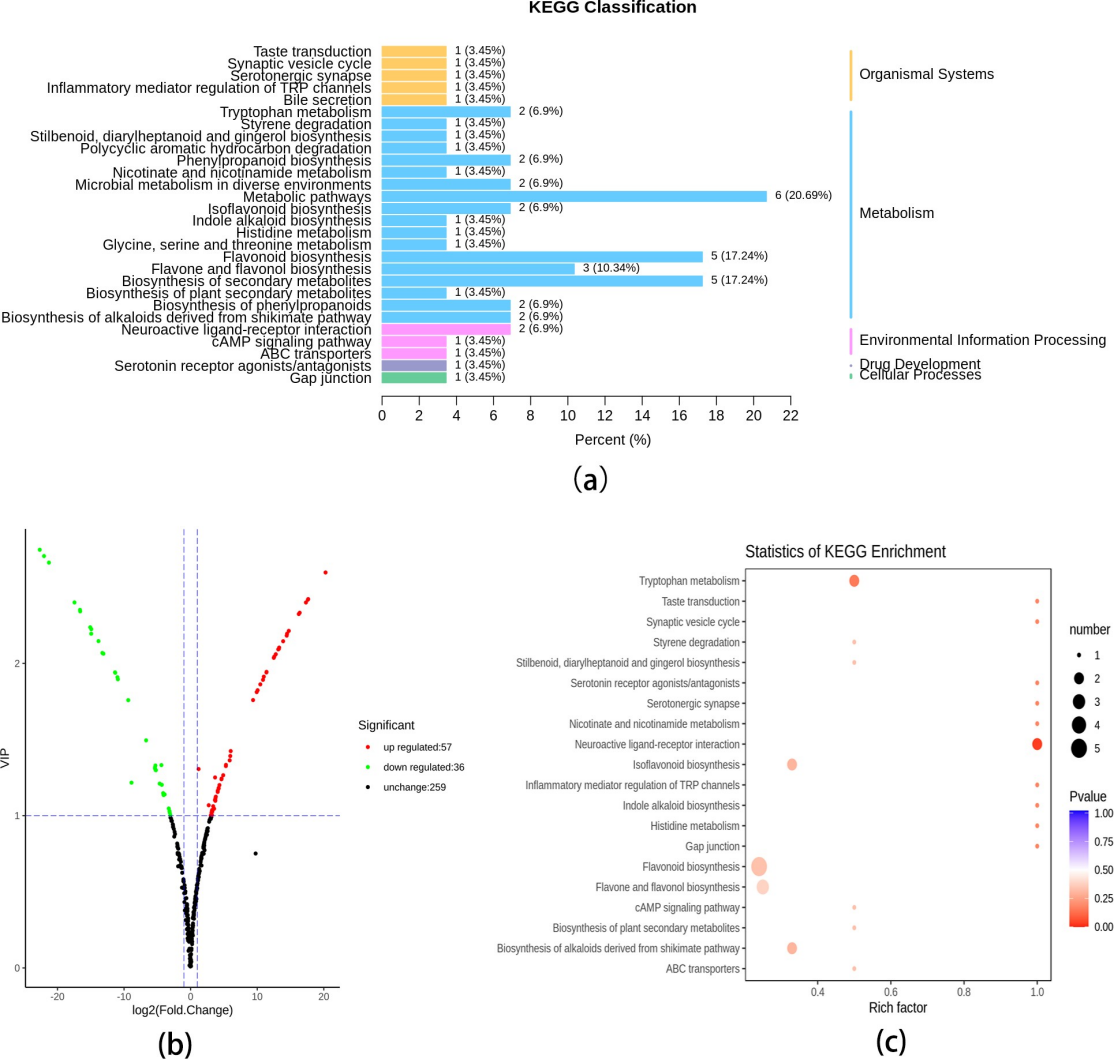
(a) KEGG classification results, (b) volcanic plots, and (c) KEGG enrichment statistics of the differential metabolites in the DHP/BZH comparison.

Differential metabolites were screened for comparison group by combining the fold change and VIP values of the OPLS-DA model. According to the criteria of fold change of values ≥2 or ≤0.5 and VIP values ≥1, the content levels of 93 metabolites (57 downregulated, 36 upregulated) are significantly different in DHP and BZH (Fig 5B). The results showed that there were more up-regulated metabolites than down-regulated metabolites in the DHP peel than that in the BZH peel. Most of the up-regulated metabolites are flavonoids. Furthermore, these differential metabolites are included in the Kyoto Encyclopedia of Genes and Genomes (KEGG) database [32]. Based on the results of KEGG classification and enrichment analysis (Fig 5A and 5C), they are mainly involved in the biosynthesis of flavonoids, flavones, flavonols, and isoflavonoids.

### Relative content comparison

**Fig 6:**
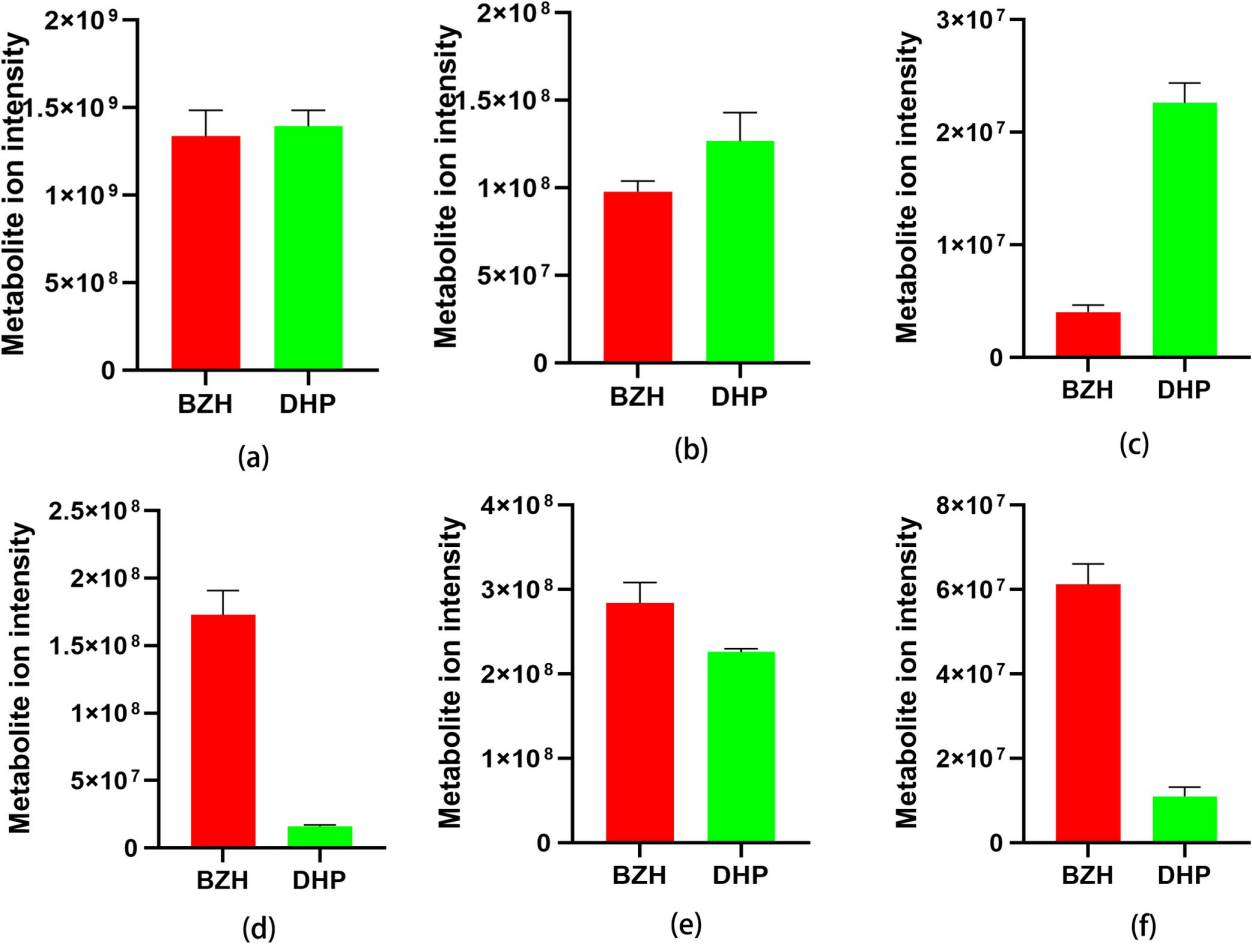
Comparision of the total ion intensity of flavonoids (a), alkaloids (b), terpenoids (c), liganans and coumarins (d), phenolic acids (e), and others (f) between BZH and DHP. Bars represent the sum of ion intensity of all metabolites belonging to each class.

To better understand the relative content of metabolites in peels of BZH and DHP, we grouped the 352 metabolites into six major classes and compared them by the metabolite ion intensity. As shown in Fig 6, citrus peels from DHP had much higher relative content of flavones, flavonoids, and flavonols than BZH did. However, the peels of BZH showed much higher relative content of liganans and coumarins, phenolic acids, and other components.

## Discussion

In this study, UPLC-ESI-MS/MS-based widely targeted metabolomics analysis was conducted to study the chemical differences between cultivars *C*. *reticulata* ‘DHP’ and *C*. *reticulata* ‘BZH’. Qualitative and quantitative UPLC-ESI-MS/MS analyses show that 352 metabolites including 11 terpenoids, 35 alkaloids, 80 phenolic acids, 25 coumarins, 7 lignans, 184 flavonoids and 10 other compounds were detected and identified, among which 15 metabolites are unique to DHP and 16 metabolites are unique to BZH. Among these unique compounds, phlorizin was studied as a potential pharmaceutical treatment for type 2 diabetes [33]; Hydroxygenkwanin has antitussive, expectorant and antiasthmatic effects [34]; Cosmosiin has anti-tumor, anti-inflammatory and anti-virus effects [35]; Avicularin has antihypertensive, antitussive and expectorant effects [36]. Those active ingredients could all be used as potential biomarkers for identifying the two cultivars. Heat-map analysis, principal component analysis (PCA), and orthogonal signal correction and partial least squares-discriminant analysis (OPLS-DA) were used to clearly discriminate between DHP and BZH, suggesting that there are significant differences in metabolic phenotypes between them.

Previously, Luo et al. [37] had identified 92 metabolites of *Citrus Reticulatae* Blanco *Pericarpium* (CRBP) and *Citrus Reticulatae* Chachi *Pericarpium* (CRCP) using ultra high-performance liquid chromatography quadrupole/time-of-flight mass spectrometry (UPLC-QTOF/MS). Of these compounds, 19 were identified as potential markers that can be used to differentiate between CRBP and CRCP. Li et al. [38] used UPLC-QTOFMS to demonstrate that different citrus cultivars can be distinguished based on 9 species-specific chemical markers including 6 flavanone glycosides and 3 polymethoxyflavones. In another study, they identified 15 chemical markers of citrus varieties using GC-MS-based metabolomics [39]. All of these reports used metabolomic methods to analyze different citrus varieties. However, these metabolomics methodologies were not validated by using QC samples. Our results suggested that the signal stability was better when the mass spectrometry was detected at different times for the same sample. At the same time, the self-built metabolite database MWDB (Metware Biotechnology Co., Ltd. Wuhan, China) and public database of metabolite information provide advantages for the extensive identification of metabolites in this paper.

Traditionally, the dried mature peel of *C*. *reticulata* ‘DHP’ is a common Chinese herbal medicine. It was reported that flavonoids, volatile components and alkaloids are the main active components. The quality was closely related to the enrichment of those active components. Compared with BZH, the peel of DHP showed much higher relative content of flavonoids, alkaloids, and terpenoids. Therefore, we suggest that the peel of BZH should not be used as a substitute for DHP.

## Conclusion

The two different citrus cultivars *C*. *reticulata* ‘DHP’ and *C*. *reticulata* ‘BZH’ were distinguished based on the widely targeted metabolomics (UPLC-ESI-MS/MS). Using this method, the two cultivars can be accurately distinguished. Overall, 352 metabolites were identified in the cultivar samples, 31 of which can be used as potential chemical markers, including 15 metabolites that are unique to DHP and 16 metabolites that are unique to BZH. Therefore, the UPLC-ESI-MS/MS-based widely targeted metabolome approach can be used to effectively differentiate between DHP and BZH. In conclusion, this study describes an effective method for the identification of closely related cultivars, especially those that cannot be distinguished based on traditional identification methods.

## Supporting information

S1 Table All the metabolites identified in this paper. (XLSX)
